# Microbial Derived Compounds Are a Promising Approach to Mitigating Salinity Stress in Agricultural Crops

**DOI:** 10.3389/fmicb.2021.765320

**Published:** 2021-11-19

**Authors:** Judith Naamala, Donald L. Smith

**Affiliations:** Smith Laboratory, Department of Plant Science, McGill University, Montreal, QC, Canada

**Keywords:** salinity, stress, microbial derived compounds, agricultural crops, plant growth

## Abstract

The use of microbial derived compounds is a technological approach currently gaining popularity among researchers, with hopes of complementing, supplementing and addressing key issues associated with use of microbial cells for enhancing plant growth. The new technology is a promising approach to mitigating effects of salinity stress in agricultural crops, given that these compounds could be less prone to effects of salt stress, are required in small quantities and are easier to store and handle than microbial cells. Microorganism derived compounds such as thuricin17, lipochitooligosaccharides, phytohormones and volatile organic compounds have been reported to mitigate the effects of salt stress in agricultural crops such as soybean and wheat. This mini-review compiles current knowledge regarding the use of microbe derived compounds in mitigating salinity stress in crops, the mechanisms they employ as well as future prospects.

## Introduction

### Soil Salinity

Soil salinity is a global problem for agricultural production, particularly in arid and semi-arid areas where crop production is significantly dependent on irrigation (Zahran, 1997, 1999; Glick, 2007; Egamberdieva and Kucharova, 2009; Egamberdieva and Lugtenberg, 2014; Shrivastava and Kumar, 2015). In the agricultural context, soil salinization refers to the accumulation of water soluble salts ions, such as Na^+^, K^+^, Mg^2+^ and Ca^2+^ and anions such as Cl^−^, ${\mathrm{SO}}_{4}^{2-}$, HCO_3_^−^, ${\mathrm{NO}}_{3}^{-}$,NO_3_^−^ and ${\mathrm{CO}}_{3}^{2-}$ (Tanji, 2002; Bui, 2013; Arora et al., 2021), in the root zone, to a level detrimental to agricultural production (Rengasamy, 2006). Na^+^, Cl^−^, Mg^2+^ and ${\mathrm{SO}}_{4}^{2-}$ ions are the most dominant in saline soils, due their high solubility, and hence, ease of deposition by water, of minerals such as NaCl, Na_2_SO_4_ and [Na_2_Mg(SO_4_)_2_] (Tanji, 2002). A soil is classified as saline when the electrical conductivity of a saturated paste soil extract (ECe) is greater or equal to 4 dS m^−1^, equivalent to 40mM NaCl (US salinity laboratory staff, 1954; Shrivastava and Kumar, 2015; Forni et al., 2017; Arora et al., 2021).

The causes of soil salinity may be natural, which results in primary salinisation, or due to human activities (anthropogenic), resulting in secondary salinization (Ghassemi et al., 1995; Tanji, 2002; Tank and Saraf, 2010; Egamberdieva and Lugtenberg, 2014; Yan et al., 2015). Geochemical weathering of minerals present in rocks is the primary natural cause (Tanji, 2002; Yan et al., 2015), although other natural factors such as: precipitation, evaporation, vegetation cover, deposition of salts from salty water bodies onto land, by wind or tsunamis, or the interactions among factors, cannot be ignored (Ghassemi et al., 1995; Tanji, 2002; Rengasamy, 2006).

On the other hand, irrigation is by far the major cause of human induced salinity, and is predominantly the cause of salinity in arid and semi-arid areas, where crop production is heavily dependent on irrigation (Rengasamy, 2006; Tank and Saraf, 2010; Rousk et al., 2011; Egamberdieva and Lugtenberg, 2014; Shrivastava and Kumar, 2015). Other anthropogenic factors, such as: application of fertilisers to the soil, deforestation and replacement of deep rooted perennial crops with shallow rooted annual crops have also contributed to soil salinisation, in one way or another (Tanji, 2002; Rengasamy, 2006; Tank and Saraf, 2010; Rousk et al., 2011; Shrivastava and Kumar, 2015). Deforestation and replacement of perennial crops with shallow rooted annual crops may result in a rising water table, thereby depositing dissolved salts in upper layers of the soil (Tanji, 2002; Tank and Saraf, 2010).

### World Spread of Soil Salinity

Climate change and excessive use of chemicals, such as fertilizers and pesticides, are major contributors to increasing soil salinity worldwide. Approximately one third of irrigated land could be rendered unsuitable for crop production due to increasing levels of soil salinization (Dodd and Perez-Alfocea, 2012). Insuficient precipitation and high rates of evapotranspiration result in soil water loss, which explains why salinity is prevalent in arid and semi-arid regions (Zörb et al., 2019). Excessive use of chemicals, such as fertilizers and pesticides, and climate change are also responsible for increasing soil salinity around the globe. In 2020, the FAO reported that, out of the 230 million hectares of irrigated land, 45 million hectares were affected by salinity stress, and that the economic effects of salinity worldwide are estimated to be about US$ 12 billion (FAO, 2020). Researchers reported that salinity affects approximately 1 billion ha of land worldwide, which represents about 7% of the planet’s surface area (Metternicht and Zinck, 2003; Yensen, 2008). In 2002, Tanji reported that saline and sodic soils cover about 10% of the total world’s arable land, although more-recent reports indicate a value as high as 50% (Yan, 2008; Xu et al., 2011). More than 100 countries worldwide have been reported to be affected by either primary or secondary salinity, or both (Tanji, 2002; Rengasamy, 2006). More than 800 million ha of land worldwide are affected by primary salinity while approximately 77 million ha are affected by secondary salinity (Ghassemi et al., 1995; Metternicht and Zinck, 2003; Yadav et al., 2011). Out of the 77 million ha affected by secondary salinity, approximately 45 million ha occur in irrigated areas (Tanji, 2002; Metternicht and Zinck, 2003). Considering that one third of the world food supply is produced on irrigated land, secondary salinization poses a very serious concern for agricultural production (Tanji, 2002). Unfortunately, due to agricultural practices and a change in climate, which has resulted in a change in rainfall patterns, evapotranspiration and landscape hydrology (Bui, 2013), soil salinization is predicted to expand at a rate of 10% annually (Shrivastava and Kumar, 2015), hence an estimated 50% of arable land is projected to be salinity affected by 2050 (Jamil et al., 2011). This high annual rate is in part attributed to the expected expansion of crop production into marginal areas, which will require irrigation (Patel et al., 2011).

## Effect of Salinity Stress on Plants

Accumulation of salt ions in the soil disrupts soil properties, such as structure, water holding capacity, pH, organic matter and nutrient content, which in turn directly or indirectly affects the capacity for plant growth in soils. Soil salinity affects plant growth and development through inducing osmotic, oxidative, and ionic stress, on the plant (Liu and Zhang, 2015; Parihar et al., 2015) As a result, plant growth at all stages, including germination, root establishment, photosynthesis, leaf area, etc. are affected, which may result in delayed maturity, as well as poor quality and quantity of yield (Parihar et al., 2015). Osmotic stress may result in reduced activity or denaturation of plant cytosolic and organelle proteins (Forni et al., 2017), decrease of cytosolic and vacuolar volumes which may negatively impact plant growth, due to reduced photosynthesis and increased production of reactive oxygen species (ROS), such as hydroxyl radicals, hydrogen peroxide, and superoxide which may be detrimental to plant cell components (Forni et al., 2017). High Cl^−^ concentrations reduce the photosynthetic capacity and quantum yield due to chlorophyll degradation and impaired photosystem II efficiency. High Na^+^ interferes with K^+^ and Ca^2+^ nutrition, affecting stomatal regulation and decreasing photosynthesis and growth. An increase in the production of ROS, over that scavenged by plant cells, results in oxidative stress, which may result in damaging of plant cells and their components such as proteins, lipids and nucleic acids (Miller et al., 2010; Del Rio, 2015). Salinity stress also affects leaf area, chlorophyll content, plant vigour, plant height, rootlength and yield quantity and quality. Plant dry matter, nutrient, metabolite and protein contents are disrupted by salinity stress (Bistgani et al., 2019; Garcia et al., 2019). Excess salt concentrations can lead to stunting and eventual death of the plants.

## Pgpm Derived Compounds For Mitigation of Salinity Stress

Plant growth promoting microbes (PGPM) exude secondary metabolites such as phytohormones, exopolysaccharides, volatile organic compounds (VOCs), and other signal molecules, that have been reported to enhance plant growth under stressed and unstressed conditions (Subramanian et al., 2016; Forni et al., 2017). The compounds mitigate the effects of abiotic stress on plants, allowing the plant to grow better than it would in the presence of stress.

**Figure 1:**
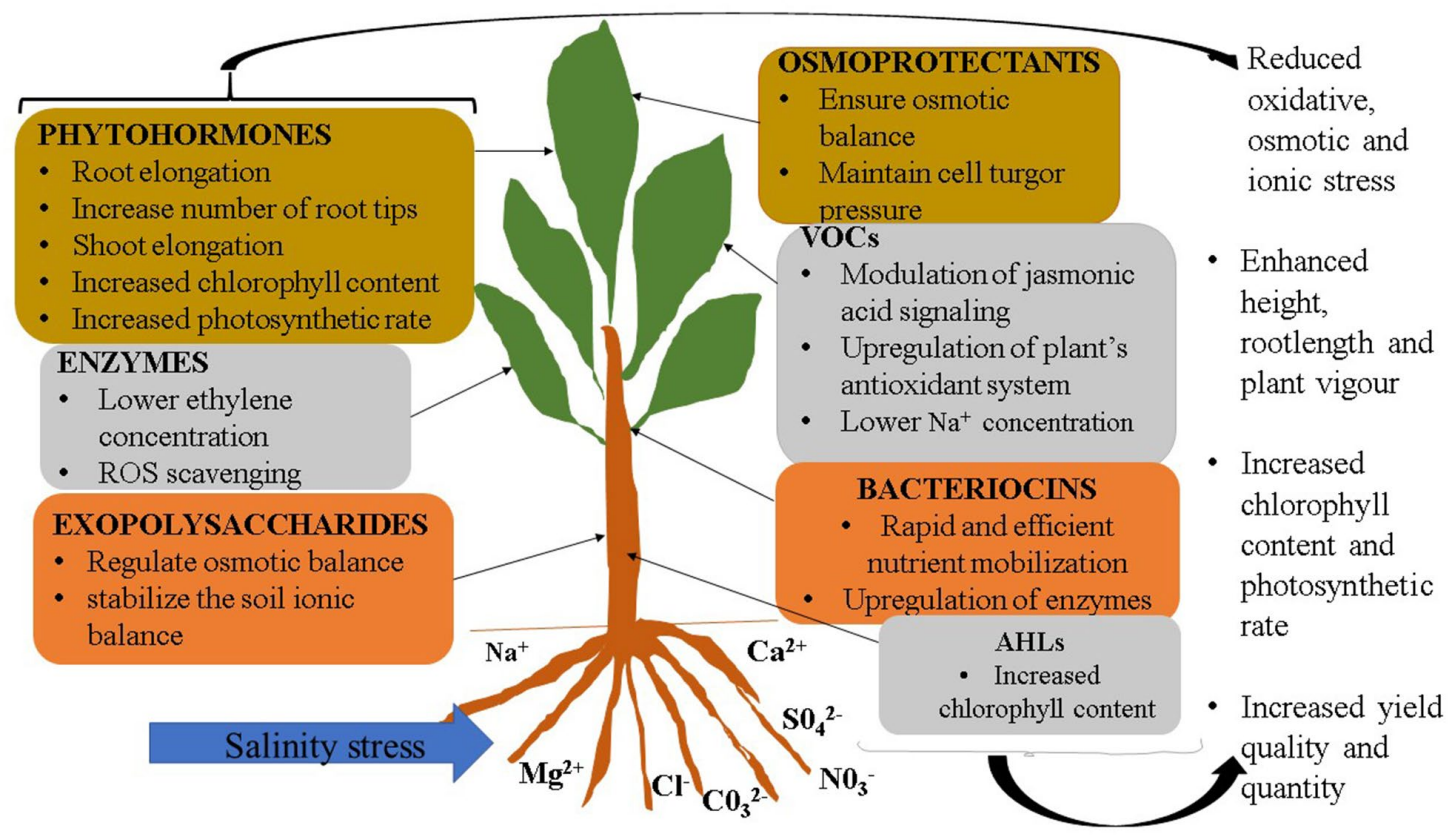
Mechanisms employed by microbial derived compounds to mitigate salinity stress in plants.

Plant growth promoting microbes can alleviate the effects of abiotic stresses, such as salinity, on plants. PGPM cells, have been reported to mitigate effects of salinity stress on plants for some time. However, results have been inconsistent, especially under field conditions. It should also be noted that excessive salt may affect growth, survival and diversity of soil microbial communities (Singleton et al., 1982), by slowing down general metabolism in cells, as well as repressing respiratory and carbon-source uptake genes (Vriezen et al., 2007; Miransari et al., 2013), which directly affects the biomass and activity of microbes (Yan and Marschner, 2012, 2013; Egamberdieva et al., 2017). Salinity causes a change in protein and metabolite structures and general morphology of the microbes, which may consequently result in loss of the microbe’s ability to promote plant growth (Zahran, 1997; Soussi et al., 2001; Tewari and Arora, 2014; Nadeem et al., 2015). In rhizobia, salinity affects the entire nitrogen fixation ability of bacteria, from root colonisation and infection to affecting the nitrogenase enzyme itself (Singleton et al., 1982; Zahran, 1997, 1999). Salinity causes a reduction in the soil water potential, which results in the flow of water out of the microbial cells into the soil, causing microbial cell drying, and lysis under severe conditions. Therefore, the idea of isolating bioactive compounds, under ideal conditions, and applying them in salt affected areas may perhaps address some of the limitations of using PGPM cells (García-García et al., 2020; Naamala and Smith, 2020, 2021; Nazari and Smith, 2020). Some microbes that produce plant growth promoting compounds can also be facultative pathogens for humans or plants, making their authorisation for use on a wider market questionable. For instance, *Pseudomonas aeruginosa* and *Bacillus anthracis* are associated with a number of human diseases (Forni et al., 2017), yet they produce high levels of compounds such as ACC deaminase. The fungus *Fusarium oxysporum* is a pathogen of many crop species, yet it produces VOCs that mitigated salinity stress effects in the model plant *Arabidopsis thaliana*, increasing chlorophyll content and leaf area (Li and Kanga, 2018). In such cases, isolating compounds in isolated and controlled conditions, from such microbes can be the most suitable way of using them to enhance plant growth, without resulting in risk to plants and humans. Use of compounds themselves may allow for control of plant exposure to such compounds, to avoid limitation associated with excess or insufficient quantities of the desired compound, something that cannot be easily done when living microbial cells are used. Additionally, PGPM derived compounds are cheaper and easier to store than PGPM cells. This would work to the advantage of farmers and agricultural product dealers that have limited storage space. A number of PGPM derived compounds have been reported to mitigate effects of salinity stress on plants; some of these are discussed below and listed and illustrated in Table 1 and Figure 1.

**Table 1:** Microbe derived compounds that have been reported to mitigate effects of salinity stress on crops.

PGPM	Compound produced	Crop species of study	References
WPR-61 (unnamed species)	ACC deaminase	*Triticum aestivum*	Arshadullah et al., 2017
*Mesorhizobium cicero*	ACC deaminase	*Cicer arietinum L*.	Brígido et al., 2013
*Bacillus amyloliquefaciens* GB03	VOCs	*Mentha piperita L*, *Arabidopsis thaliana*	Cappellari et al., 2020; Zhang et al., 2008a
*Pseudomonas extremorientalis* TSAU20	IAA	*Silybum marianum*	Egamberdieva et al., 2013
*Pseudomonas aure-antiaca* TSAU22	IAA	*Triticum aestivum*	Egamberdieva, 2009
*Pseudomonas putida* UW4	ACC deaminase	*Cucumis sativus*	Gamalero et al., 2010
*Bacillus thuringiensis*	Thuricin17	*Glycine max*, *Zea mays* and *Brassica napus*	Gautam et al., 2016; Schwinghamer et al., 2016; Subramanian et al., 2016
*Bradyrhizobium japonicum*	LCO	*Glycine max*	Gautam et al., 2016; Subramanian et al., 2016
*Trichoderma koningii*	VOCs	*Arabidopsis thaliana*	Jalali et al., 2017
*Fusarium oxysporum*	VOCs	*Arabidopsis thaliana*	Li and Kanga, 2018
*Verticillium dahliae*	VOCs		Li and Kanga, 2018
*Bacillus amyloliquefaciens*	VOCs	*Arabidopsis thaliana*	Liu et al., 2020
*Bradyrhizobium japonicum*	LCO	*Zea mays*	Nandhini and Somasundaram, 2018
*Bacillus amyloliquefaciens*	Abscisic acid, gibberellins	*Oryza sativa*	Shahzad et al., 2016, 2017
*Pseudomonas fluorescens*	ACC-deaminase	*Arachis hypogea*	Saravanakumar and Samiyappan, 2007
*Burkholderia graminis*,	AHLs	*Solanum lycopersicum*	Barriuso et al., 2008

### Phytohormones

Plant growth promoting microbes exude phytohormones such as auxins, cytokinins, jasmonates, etc., which have been reported to enhance various aspects of plant growth, such as root length, number of root tips, shoot elongation, plant fresh and dry weight, increased chlorophyll content and photosynthetic rate, etc. (Spaepen and Vanderleyden, 2011; Belimov et al., 2015; Forni et al., 2017). This improves nutrient uptake and consequently improves plant yield quality and quantity under stressed and non-stressed conditions (Khan et al., 2019). In plants, phytohormones like abscisic acid (ABA) and jasmonic acid (JA) can be produced in response to stress, to protect plants from the effects of ROS and the resulting oxidative stress (Cappellari and Banchio, 2020). ABA and JA also play a role in many stress signaling pathways. Ethylene is also a phytohormone produced by plants in response to stress factors. However, excessive amounts of ethylene can be detrimental to plant development, resulting in reduced plant growth. The most studied phytohormones are IAA, ethylene, salicylic acid, ABA and jasmonic acid. The role of microbial phytohormones in mitigating salt stress on various plants has been reported by researchers (Egamberdieva, 2009; Bianco and Defez, 2009; Egamberdieva et al., 2013; Tewari and Arora, 2014; Egamberdieva et al., 2015; Forni et al., 2017). Strain *Curtobacterium* sp. SAK1 enhanced salt stress tolerance in soybean (*Glycine max* cv. Pungsannamu) through production of phytohormones gibberellins, IAA, and ABA (Khan et al., 2019), among other mechanisms.

### Enzymes

Microbial derived enzymes, such as 1-aminocyclopropane-1-carboxylate (ACC) deaminase play a significant role in mitigating the effect of salinity stress on plants. Ethylene is an essential phytohormone, required by plants at certain stages of development such as germination and ripening. However, when in excess, which is usually the case when plants encounter stress, it becomes detrimental to plant growth by promoting leaf abscission and inhibition of root elongation. The enzyme ACC deaminase lowers ethylene concentration by breaking down ACC, the precursor of ethylene, into ammonium and an energy source, alpha-keto butyrate (Glick et al., 1998; Burd et al., 2000; Tank and Saraf, 2010; Ali et al., 2014; Pérez-Montano et al., 2014; Jha and Saraf, 2015; Nadeem et al., 2015; Khan et al., 2019). Consequently, the effect of excess ethylene, that result from stress, are mitigated by the enzyme ACC deaminase. The effect of ACC deaminase in mitigating effects of salinity stress in various crop species has been reported (Mayak et al., 2004b; Saravanakumar and Samiyappan, 2007; Gamalero et al., 2010; Kasotia et al., 2012; Brígido et al., 2013; Khan et al., 2019). ACC-deaminase produced by *Pseudomonas fluorescence* enhanced salt tolerance in groundnut (Saravanakumar and Samiyappan, 2007) while ACC deaminase producing *Curtobacterium* sp. SAK1 mitigated salinity stress in soybean (Khan et al., 2019).

Other enzymes produced by PGPM, such as s catalases, superoxide dismutase and ascorbate peroxidase are antioxidants that degrade ROS such as peroxides (Lopes et al., 2021).

### Osmoprotectants

Salt stress imposes osmotic and ionic stress effects on affected plants which in turn affect plant water uptake and general growth and development. Some PGPMs have been reported to produce osmoprotectants such as proline, glycerol, glutamate, glycine and trehalose (Suarez et al., 2008; Rodriguez-Salazar et al., 2009; Forni et al., 2017). Osmoprotectants are metabolites or compatible solutes with a low molecular weight, which minimise osmotic and ionic stress by controlling stomata opening and transpiration rate, maintaining plant cell turgor pressure and equal ion efflux across the plant cell membrane (Dodd and Perez-Alfocea, 2012; Paul and Lade, 2014; Saghafi et al., 2019), hence, enhancing plant growth under salt stress conditions. For instance, proline has been reported to mitigate effects of salinity stress in sweet pepper, cowpea, rice and tomato (Roy et al., 1993; Heuer, 2003; Hossain et al., 2011; Abdelaal et al., 2020).

### Volatile Organic Compounds

Microbial VOCs have low boiling points and low molecular masses, of approximately 300Da and below (Veselova et al., 2019; Weisskopf et al., 2021). Microbial VOCs are emitted as secondary metabolites, at different growth stages and under different growth conditions (Fincheira et al., 2021). The major volatiles produced by microbes include: alkenes, alcohols, ketones, terpenes, benzenoids, pyrazines, acids, esters and aldehydes (Poveda, 2021). Microbial VOCs have been reported to enhance growth of plants exposed to salinity or osmotic stress (Timmusk et al., 2014; Jalali et al., 2017). For instance, *Arabidopsis thaliana* plants treated with VOCs from *Trichoderma koningii* tolerated 100mM NaCl (Jalili et al., 2016). *Pseudomonas simiae* VOCs 4-nitroguaiacol and quinoline enhanced tolerance of soybean to 150mM salt stress (Vaishnav et al., 2016). VOCs produced by *Pseudomonas simiae* strain AU mitigated effects of salt stress in soybean plants by reducing Na^+^ and increasing phosphorus and potasium concentration (Vaishnav et al., 2015). Furthermore, VOCs produced by *Bacillus amyloliquefaciens* GB03 increased essential oil yield in *Mentha piperita* L. exposed to 0, 75 and 100mM NaCl. Interestingly, the microbial VOCs are biodegradable, nontoxic and are required at lower concentrations to stimulate plant growth (Fincheira et al., 2021), which is makes VOCs desirable approaches in the PGPM technology.

### Exopolysaccharides

Microbial exopolysaccharides are diverse, varying in function and structure and differ across microbial species. The concentration of EPS produced also differs across species (Bhagat et al., 2021). Their structure is complex, comprising biomolecules such polysaccharides, structural proteins, enzymes, amino sugars, nucleic acids, lipids, pyruvates, glycoproteins, etc. (Mishra and Jha, 2013). PGPM produce exopolysaccharides for reasons such as, aiding microbial attachment to plant roots and formation of biofilms (Ruppel et al., 2013; Qin et al., 2016; Liu et al., 2017b; Bhagat et al., 2021). EPS bind with cations, such as Na^+^, lowering their bioavailable ions and hence, plant uptake, creating osmotic balance and stabilizing the soil ionic balance, thus, mitigating osmotic and oxidative stress in plants (Kumar et al., 2020; Lopes et al., 2021). Microbial exopolysaccharides have been reported to mitigate the effect of salinity stress on different crop species such as maize, mung bean and wheat (Ashraf et al., 2004; Rojas-Tapias et al., 2012; Mahmood et al., 2016). For instance, EPS produced by *Azotobacter chrococcum* strains alleviated effects of salt stress in maize (Rojas-Tapias et al., 2012). Lipochitooligosaccharide is a signal molecule produced by rhizobia to communicate with its host plant. The same molecule, isolated from *Bradyrhizobium japonicum* has been reported to enhance plant growth under saline conditions (Gautam et al., 2016; Subramanian et al., 2016; Nandhini and Somasundaram, 2018). Soybean seeds treated with LCO upregulated proteins essential for salt stress tolerance (Subramanian et al., 2016).

### *N-Acyl*-Homoserine Lactones

*N-Acyl*-Homoserine Lactones (AHLs) are quorum sensing signals mostly produced by gram negative bacteria, to communicate and coordinate population behavior such as siderophore production and biofilm formation (Zhao et al., 2020). They consist of a conserved homoserine lactone (HL) ring and an amide (*N*)-linked acyl side chain which is either saturated or non-saturated (Ortiz-Castro and Lopez-Bucio, 2019). A microbe can posses more than one AHL quorum sensing system. There are a number of quorum sensing molecules with both short and long carbon chains, with a carbon chain range of 4–18 carbons. Research findings show that plants can absorb AHLs through the root system and translocate them to the shoot (Sieper et al., 2014). Researchers have reported the ability of quorum sensing signals to enhance salt tolerance in plants. For instance, N-3-oxo-hexanoyl-homoserine lactone (3OC6-HSL) enhanced rootlength, chlorophyll content and fresh weight in wheat and arabidopsis exposed to salt stress (Zhao et al., 2020). Long-chained AHL compounds produced by *Burkholderia graminis*, were reported to enhance both growth and salt tolerance in tomato (Barriuso et al., 2008). Nawaz et al., 2020 reported an increase in measured root variables of both salt sensitive and salt tolerant wheat cultivars inoculated with AHLs from *Aeromonas* spp. Therefore, AHLs are a promising approach to mitigation of salinity stress effects in agricultural crops.

### Bacteriocins

Plant growth promoting microbes, especially bacteria, produce bacteriocins for various purposes such as signaling and for competition against related bacterial species (Smith et al., 2015; Subramanian and Smith, 2015; Nazari and Smith, 2020). Bacteriocins are synthesised in the ribosome of bacteria, majorly, as antimicrobials against closely related microbes (Arnison et al., 2013; Mak, 2018). There are different classes of bacteriocins, depending in part on whether they are produced by gram positive or gram negative bacteria. For instance, Chavan and Riley (2007) indicate that gram negative bacteriocins are classified as colicins, colicin-like bacteriocins, microcins, and phage tail-like. Bacteriocins from gram positive bacteria are classified as class I (sub classes Ia and Ib), class II (subclasses IIa, IIb, IIc and IId) and class III (Nazari and Smith, 2020). Although bacteriocins are mostly known for their antimicrobial properties,, recent studies show that they are able to act as microbe-to-plant signals enhancing growth and salt tolerance in a wide range of plant species. In this perspective, thuricin 17, a bacteriocin produced by the gram positive *Bacillus thuringiensis* NEB17 is the most studied, in relation to promotion of plant growth (Subramanian and Smith, 2015; Nazari and Smith, 2020). Subramanian et al. (2016) reported that thuricin 17 enhanced tolerance of soybean to up to 150mM NaCl stress. *Arabidopsis thaliana* treated with thuricin 17 tolerated up to 250mM NaCl (Subramanian, 2014). Canola plants treated with 0.2M NaCl and 10^−9^M thuricin 17 were taller than plants treated with just 0.2M NaCl (Schwinghamer et al., 2016).

## Mechanisms Through Which Microbe Derived Compounds Enhance Plant Tolerance To Salinity Stress

There are various mechanisms through which microbe derived compounds mitigate effects of salinity stress on plants. Most of the mechanisms aim at alleviating the three major effects of salt stress, i.e., oxidative, osmotic and ionic stress. However, some mechanisms, such as increased chlorophyll content aim at increasing photosynthesis and subsequent plant growth. Upregulation of the plant’s anti-oxidation system, through upregulation of anti-oxidation enzymes is one of the mechanisms exhibited by microbe derived compounds. Such enzymes include, peroxidases, catalases, superoxide dismutase, glutathione reductase and ascorbate peroxidase (Bianco and Defez, 2009; Forni et al., 2017). Some compounds have been reported to activate synthesis of plant genes that encode anti-oxidation enzymes (Timmusk et al., 2014), as a means of upregulating the plant’s anti-oxidation system. Microbe derived VOCs increased concentration of DPPH (2,2-diphenyl−1-picrylhydrazyl) radical scavenging activity while lowering concentration of malondialdehyde (MDA) levels in pepper mint (Cappellari et al., 2020).

Improvement of ion homeostasis is another mechanisms through which compounds mitigate salinity stress in plants. This mechanisms works to eliminate osmotic and ionic stress in salt affected plants. The compounds regulate uptake of compounds and or ions such as proline, K^+^ and Na^+^. For instance, some compounds such as VOCs and ACC deaminase have been reported to increase K^+^ uptake and root to shoot K^+^ flow while lowering Na^+^ uptake, in plants such as *Arabidopsis thaliana* and soybean (Mayak et al., 2004a; Zhang et al., 2008a; Vaishnav et al., 2015). For instance, *Pseudomonas simiae* strain AU VOCs decreased root Na^+^ levels and increased the accumulation of the osmoprotectant proline, in soybean (Vaishnav et al., 2015). *Bacillus amyloliquefaciens* FZB42 VOCs not only decreased the Na^+^ contents of the whole plants but also induced the expression of genes such as HKT1; high-affinity K^+^ transporter, that function to alleviate Na^+^ toxicity (Liu et al., 2020). Upregulation of ion-homeostasis regulation genes SOS1, SOS2 and SOS3 by microbial VOCs was reported in salt stressed *Arabidopsis* (Zhao et al., 2020).

Upregulation of salt responsive genes such as *COR15a*, *RD22*, *ADH*, *P5CS1* and *ERD1* was reported (Zhao et al., 2020). Modulation of jasmonic acid signaling was also reported as a mechanism employed by *Bacillus amyloliquefaciens* FZB42 VOCs, in alleviating salinity stress (Liu et al., 2020). AHL 3OC6-HSL increased chlorophyll content in wheat and *Arabidopsis thaliana* (Zhao et al., 2020). Lowering ethylene concentration is a mechanism employed by ACC-deaminase. Production of siderophores and phosphorus solubilisation.

Soybean salt stressed seeds treated with thuricin17 exhibited more rapid and efficient mobilization of carbon, nitrogen, and storage proteins which resulted in enhanced germination than controls (Subramanian et al., 2016a). In salt stressed *Arabidopsis thaliana*, treatment with thuricin17 resulted in alteration of carbon and energy metabolism pathways and upregulation of PEP carboxylase, rubisco-oxygenase, pyruvate kinase, and proteins of the light harvesting complex, energy and antioxidant pathways (Subramanian et al., 2016b).

## Challenges and Way Forward

Since this is a relatively new technology, there is currently limited knowledge on the technology of using microbial derived compounds, in general. Hence, there are limited compounds on the market, that have been reported to eliminate salt stress in agricultural crops. Moreover, isolation of some compounds is a complicated and time consuming process that many companies may not be willing to get involved in. There is a lot to be learned, concerning concentration, formulation, shelf life and mode of application of microbial derived compounds. Further still, majority of studies have been conducted in greenhouses and growth chambers, with limited findings under field conditions, yet, effectiveness of microbial technology is known to be inconsistent under field conditions.

There is need for more studies on microbial derived compounds and salinity stress, especially under field conditions, to address all the above mentioned challenges.

## Author Contributions

NJ gathered literature and wrote the mini-review. SD advised on grammar and scientific knowledge, and provided the intellectual context. All authors contributed to the article and approved the submitted version.

## Funding

This work was funded through a grant from Consortium de recherche et innovations en bioprocédés industriels au Québec, number CRIBIQ 2017-034-C30.

## Conflict of Interest

The authors declare that the research was conducted in the absence of any commercial or financial relationships that could be construed as a potential conflict of interest.

## Publisher’s Note

All claims expressed in this article are solely those of the authors and do not necessarily represent those of their affiliated organizations, or those of the publisher, the editors and the reviewers. Any product that may be evaluated in this article, or claim that may be made by its manufacturer, is not guaranteed or endorsed by the publisher.
